# An analysis on the relationship between safety awareness and safety behaviors of healthcare professionals, Ankara/Turkey

**DOI:** 10.1002/1348-9585.12129

**Published:** 2020-06-23

**Authors:** Fatma Uzuntarla, Serhat Kucukali, Yasin Uzuntarla

**Affiliations:** ^1^ Department of Occupational Health and Safety Cankaya University Ankara Turkey; ^2^ Department of Civil Engineering Hacettepe University Ankara Turkey; ^3^ Department of Health Management Gülhane Training and Research Hospital Ankara Turkey

**Keywords:** healthcare professionals, safety awareness, safety behaviors

## Abstract

**Objectives:**

This descriptive study aims to examine the relationship between the safety awareness of healthcare professional and their safety behaviors.

**Methods:**

The study was carried out on 418 healthcare professionals working in a training and research hospital in Ankara/Turkey. The survey method was used as data collection tool. The questionnaire consisted of 3 sections and 18 questions. First section consisted of questions on sociodemographic characteristics and, second section consisted of the awareness scale and third section consisted of safety behaviors scale.

**Results:**

The safety awareness and safety behaviors are scored on a scale from 1 (completely disagree) to 5 (completely agree). The safety awareness and safety behaviors has an average score of 3.85 ± 0.81 and 3.56 ± 0.82, respectively. The safety awareness and safety behavior levels of healthcare professionals were found to be high.

**Conclusion:**

A significant positive correlation was found between safety awareness and safety behaviors and it was concluded that the increase in safety awareness led to an increase in safety behavior.

## 1 INTRODUCTION

Employees spend a significant part of their time in workplaces. Therefore, occupation‐related tasks need to be at a level that will not be harmful to health of the employees and the work should be performed in a safe environment.^1^ However, occupational accidents and occupational diseases, especially in the underdeveloped and developing countries, are still high. This leads to death, disability, economic and social loss, and thus attention on the importance of occupational health and safety.^2^

Despite the numerous legal regulations governing the prevention of occupational accidents within the context of occupational health and safety, occupational accidents are still high. This has shown that it is not enough to consider this issue only from a technical point of view, but individuals being most important factor in occupational accidents should also be taken into consideration. The fact that approximately 90% of occupational accidents are caused by unsafe behaviors of employees highlights the importance of ‘safety culture’, regarded as a behavioral regulator.^3^, ^4^, ^5^

Safety culture (SC) is the ability of an organization to put safety rules into practice and successfully manage these in all of the activities and operations.^6^ SC was first mentioned in the report prepared after the explosion of the Chernobyl Nuclear Power Plant in Chernobyl, Ukraine, in 1986. Studies have shown that safety culture is actually a subdimension and the reflection of organizational culture.^7^, ^8^ The belief that safety culture significantly effects health and safety increases in addition to the studies on this topic. Factors that constitute SC can be classified as safety priorities, safety communication, safety training, employee participation, fatalism, and safety awareness.^9^

Safety awareness (SA) is the frame of the mind that determines the perceptions and judgments of employees about personal abilities and responsibilities to avoid risks in workplaces.^10^ Safety behavior (SB) is the behavior of an individual by safety policies and procedures.^11^

Hospitals are institutions with hazardous jobs and duties because of their varied specialties, job process, and intense technology usage. Healthcare personnel are always faced with work accident and occupational disease while providing healthcare service. Such dangers in the hospital environment cause a threat toward both health personnel and patients, also leads to medical failures. So, protecting health personnel from these dangers also means protecting patients form medical failures. In order to minimize the potential risks, it is necessary to comply with the accreditation standards in healthcare institutions, to apply quality management systems, to conduct research that reveals the current situation regarding employee health and safety, and to raise awareness of health professionals.^12^, ^13^, ^14^, ^15^ When reviewing the literature, such research is very rare and especially in the healthcare sector in Turkey. So, this research is thought to contribute necessary information in this gap.

In the literature, there are some studies that demonstrate the relationship between safety awareness and occupational safety‐related behaviors in different sectors such as: textile and metal sectors in Turkey,^7^ food sector in Slovenia,^9^ and manufacturing sector in Turkey.^10^ This study aims to examine the relationship between the safety awareness and safety behaviors of healthcare professionals.

## 2 MATERIALS AND METHODS

The research was carried out at Gülhane Training and Research Hospital between January and March 2018. Gülhane Training and Research Hospital was one of the most modern tertiary hospitals of the country with 1200 bed capacity, located on Ankara, capital of the Turkey. The hospital currently provides healthcare service to all citizens under the Ministry of Health, once was the country's largest military hospital between 1898 and 2016, served military personnel. In this study, the sample was not taken, instead, aimed at surveying with all personnel face to face. However, some of the hospital employee could not be reached due to reasons such as permission, patient leave, assignment, and not wanting to participate in the survey. Accordingly, the survey response rate was 35% and 418 healthcare professionals participated to the survey in line with the Helsinki Declaration code of ethics.

### 2.1 Questionnaire

Survey method was used as data collection tool in this study. The questionnaire consisted of 3 sections and 18 questions.

#### 2.1.1 Sociodemographic characteristics

Seven questions were included in this section based on a literature review conducted by the authors: Age, gender, marital status, educational status, monthly income level of the participants, occupation, and term of employment.

#### 2.1.2 Safety awareness scale (SAS)

SAS developed by Lin et al and adapted to Turkish by Dursun consists of five questions. SAS being a 5‐point Likert‐type scale has “Completely disagree” scored as 1 and “Completely agree” was scored as 5 points. High scores indicate a high level of occupational safety awareness.^3^, ^16^

#### 2.1.3 Safety behavior scale (SBS)

Developed by Neal, Griffin, and Hart and adapted to Turkish by Dursun, SBS consists of two subdimensions as safety compliance (SC) and safety participation (SP) and a total of six questions. SBS is a 5‐point Likert‐type scale. “Completely disagree” is scored as 1 and “Completely agree” is scored as 5 points. High scores indicate a high level of safety behavior.^3^, ^17^ SC refers to the compliance of personnel to safety principles and rules, and act by safety processes. SP refers to the voluntary participation of personnel in activities, training, and meetings related to safety.^18^, ^19^

### 2.2 Statistical analysis

SPSS (Version 22, Chicago, IL, USA) statistical program was used to evaluate the research data. The arithmetic mean, standard deviation, and frequency analysis were used to analyze descriptive statistics. Kolmogorov‐Smirnov test was used to determine whether research variables’ data were normally distributed. Since research variables’ data were normally distributed (Kolmogorov‐Smirnov test; *P* > .05), multiple linear regression analysis with stepwise method was used to test the relationship between variables. In this multiple linear regression analysis, as a stepping method, probability of F was used with entry threshold: 0.005 and removal threshold 0.010. SBS mean score was taken to the regression model as a dependent variable. SC and SP subdimensions were excluded from the regression model to prevent multicollinearity. SAS mean score, age, gender, marital status, educational status, monthly income level, occupation, and tenure were taken to the regression model as an independent variable. Ordinal sociodemographic characteristics, educational status, monthly income level, and tenure were put into the regression model as they are, however, categorical sociodemographic characteristics, gender, marital status, and occupation, were put in the regression model after converted into dummy variables. *P* < .05 was accepted as a significant level.

## 3 RESULTS

Reliability coefficients of the scales were found as 0.86 for SAS, 0.88 for SBS, 0.90 for SC, and 0.82 for SP. Therefore, the scales used in the research are highly reliable. A total of 418 healthcare personnel participated in our research and their sociodemographic characteristics were presented in Table 1. Participants' ages ranged from 18 to 60 years old, with an average of 37.1 ± 8.5 years. Of the participants, 223 (53.3%) were females and 195 (46.7%) were males. Of the participants, 57 (13.6%) were physicians, 150 (35.9%) were nurses, 98 (23.5%) were health technicians, 26 (6.2%) were technical personnel, and 87 (20.8%) were other health personnel.

**TABLE 1:** Sociodemographic characteristics of the participants

Variable		n(%)
Age (y)	<30	171 (40.9)
	31‐40	139 (33.3)
	>40	108 (25.8)
Gender	Female	223 (53.3)
	Male	195 (46.7)
Marital status	Married	261 (62.4)
	Bachelor	157 (37.6)
Educational status	Primary education	16 (3.8)
	High school	25 (6.0)
	University	282 (67.5)
	MSc.	58 (13.8)
	PhD./ Specialist MD.	37 (8.9)
Monthly income	<875$	125 (29.9)
	875‐1500$	149 (35.7)
	>1500$	144 (34.4)
Occupation	Physician	57 (13.6)
	Nurse	150 (35.9)
	Health technician	98 (23.5)
	Technical personnel	26 (6.2)
	Other health personnel	87 (20.8)
Tenure (y)	<6	152 (36.4)
	6‐10	69 (16.5)
	>10	197 (47.1)

Scales had the following mean and standard deviation values, respectively: 3.85 ± 0.81 for SAS, 3.56 ± 0.82 for SBS, 3.59 ± 0.95 for SC subdimension, and 3.53 ± 0.88 for SP subdimension. Mean and standard deviation of the scales and subscales were presented along with percentage and frequency participants answers to scale items in Table 2. Participants' safety awareness, safety behaviors, safety compliance, and safety participation levels were found to be high.

**TABLE 2:** Distribution and mean of participants’ responses to SAS, SBS, SC, and SP items

Scales		M ± SD	Completely disagree	Disagree	Undecided	Agree	Completely agree
			n (%)	n (%)	n (%)	n (%)	n (%)
SAS (3.85 ± 0.81)	1. I am clear about what my responsibilities are for the workplace safety	3.82 ± 1.03	22 (5.3)	27 (6.5)	53 (12.7)	215 (51.4)	101 (24.2)
	2. I understand the safety rules for my job	3.95 ± 0.98	17 (4.1)	24 (5.7)	37 (8.9)	221 (52.9)	119 (28.5)
	3. I can deal with safety problems at my workplace	3.55 ± 1.05	16 (3.8)	59 (14.1)	93 (22.2)	177 (42.3)	73 (17.5)
	4. I comply with the safety rules all the time	3.73 ± 1.02	13 (3.1)	46 (11.0)	72 (17.2)	195 (46.7)	92 (22.0)
	5. When I am at work, I think safety is the top important thing	4.17 ± 1.00	15 (3.6)	23 (5.5)	21 (5.0)	173 (41.4)	186 (44.5)
SBS (3.56 ± 0.82							
SC (3.59 ± 0.95)	1. I use all necessary safety equipment to do my job	3.60 ± 1.06	18 (4.3)	60 (14.4)	63 (15.1)	204 (48.8)	73 (17.5)
	2. I use the correct safety procedures for carrying out my job	3.73 ± 1.01	15 (3.6)	44 (10.5)	63 (15.1)	209 (50.0)	87 (20.8)
	3. I ensure the highest levels of safety when I carry out my job	3.44 ± 1.04	20 (4.8)	64 (15.3)	94 (22.5)	188 (45.0)	52 (12.4)
SP (3.53 ± 0.88)	4. I promote the safety program within the organization	3.59 ± 1.02	17 (4.1)	52 (12.4)	81 (19.4)	203 (48.6)	65 (15.6)
	5. I put in extra effort to improve safety of the workplace	3.51 ± 1.03	17 (4.1)	61 (14.6)	89 (21.3)	193 (46.2)	58 (13.9)
	6. I voluntarily carry out tasks or activities that help to improve workplace safety	3.50 ± 1.02	19 (4.5)	56 (13.4)	93 (22.2)	196 (46.9)	54 (12.9)

According to findings of the multilinear regression model, statistically significant relationships were found between safety behaviors and safety awareness (F: 593.378; *P* < .001). It was also seen that 58% of variance change in safety behaviors mean was explained by safety awareness (R^2^:0.58). Additionally, increasing safety awareness raises safety behavior positively as well (β: 0.767; *P* < .001) (Table 3). However, we found no significant relationships between safety behavior and other independent variables (*P* > .05). Accordingly, those independent variables were excluded from the model.

**TABLE 3:** Multiple linear regression model of relationships between safety awareness and safety behavior

Regression Model Summary			
R	R Square	f	Sig
0.767	0.588	593.378	<0.001

Coefficients^a^						
Model		Unstandardized Coefficients		Standardized Coefficients	t	Sig.
		B	Std. Error	Beta		
1	(Constant)	0.589	0.125		4.711	<0.001
	Safety awareness	0.773	0.032	0.767	24.359	<0.001
*a. Dependent Variable:* Safety behavior						

^a^ Dependent Variable: Safety behavior.

## 4 DISCUSSION

Previous studies on safety awareness and safety behavior levels have been carried out on employees in specific industries such as manufacturing, occupational safety, telecommunication, food, construction, metal, and petrochemical.^3^, ^4^, ^8^, ^9^, ^19^, ^20^, ^21^ This study is unique and important because it is the first study to be conducted in the healthcare sector in Turkey.

The reliability coefficient of SAS used in this study was found to be 0.83 in the original paper and 0.81‐0.90 in similar studies. In the original paper, reliability coefficients of SC and SP were 0.94 and 0.85, respectively, and these values were between 0.80‐0.95 in similar studies. Our reliability findings were found to be consistent with the results of other studies, which had similarly reliability coefficient higher than 0.80.^3^, ^16^, ^17^, ^19^, ^20^, ^21^ When the reliability coefficients of the scales were examined, they were found in our study as well as in other studies. We can conclude that the scales are highly reliable and can be used in future studies.

When the mean values of the scales used in our study were examined, it was found that mean values of 3.44‐4.17 points (1‐5 points) were obtained and these results were consistent with the results of other studies.^9^, ^20^, ^21^ Therefore, it is evaluated that the safety awareness, exhibited safety behaviors, safety compliance, and safety participation levels of healthcare professionals participating in our study are high. In the context of occupational health and safety studies, the fight against occupational accidents is at the forefront and ignoring occupational safety leads to occupational accidents. Occupational accidents in the healthcare sector occur 34% more compared to the figures in other sectors and it shows how important this topic is for the healthcare sector. When the causes of occupational accidents are examined, it is seen that lack of training and experience, and the presence of inappropriate working conditions leading to accidents are among the top causes.^22^, ^23^, ^24^ It is highly important to improve working conditions, and to raise awareness of personnel by providing training events in order to prevent and decrease occupational accidents.

There was no significant relationship between sociodemographic characteristics and safety behaviors in our study. Some previous studies reported that men presented more safety behaviors than women, and married individuals exhibited more safety behaviors than single individuals, and individuals with less occupational experience presented more safety behaviors than individuals with more occupational experience.^21^, ^25^, ^26^ While some of the previous studies did not find a significant relationship between sociodemographic characteristics and safety awareness, some studies found that the middle/elderly age group had higher awareness than the younger age group, and women had higher awareness than men.^27^, ^28^, ^29^, ^30^, ^31^

In our study, there is a significant relationship between safety awareness and safety behaviors, and as safety awareness increases, the level of safety behaviors also increases. Furthermore, safety awareness explains 58% of safety behaviors. The results of our study are consistent with previous studies and we propose that focusing on safety awareness will contribute to increasing safety behavior levels.^32^, ^33^, ^34^, ^35^, ^36^, ^37^, ^38^

### 4.1 Limitations

This research was limited to the healthcare professionals working at Gülhane Training and Research Hospital (Ankara/Turkey), findings of which cannot be generalized with this low participation rate.

The other limitation of this research is that safety behaviors of the participants were measured with their statements in the survey.

## 5 CONCLUSION

This study aimed to examine the relationship between the safety awareness and safety behaviors of healthcare professionals.

It was found that the SAS and SBS scales used in our study were highly reliable and could be used in future studies. It was observed that the safety awareness, exhibited safe behaviors, safety compliance, and safety participation levels of healthcare professionals participating in the study were high. A positive and statistically significant correlation was found between safety awareness and safety behaviors. It has been concluded that an increase in safety awareness leads to an increase in safety behavior levels.

Topics related to the importance and priority of occupational safety should be covered in educational curricula and subsequently in orientation and in‐service training after graduation to increase the culture and awareness of occupational safety. Thus, safer working conditions will be achieved through personnel with adequate occupational safety awareness upon graduation instead of increasing safety awareness while working and awareness will be further increased by current training events after graduation. Furthermore, when the literature is examined it is seen that studies related to this topic are mainly carried out in the manufacturing and construction sectors. Studies in the healthcare sector are not very common. It is recommended that more studies should be performed in this field.

## DISCLOSURES

*Approval of the research protocol:* The necessary permissions were obtained from Cankaya University Ethics Committee (2017/102) and Gulhane Training and Research Hospital Scientific Research Commission (2017/12) for this study. *Informed consent:* Informed written consent was obtained from each participant before starting the study. *Registry and the registration no. of the study:* N/A. *Animal studies:* N/A. *Conflict of interest:* The authors declare no conflict of interests.

## AUTHOR CONTRIBUTIONS

FU participated in the design and conception of the study and its coordination, acquisition of data, carried out statistical analysis, and drafted the manuscript. SK participated in the conception of the study and participated in the design of the study, and reviewed analysis and manuscript. YU participated in the design of the study, acquisition of data, and performed the statistical analysis.
